# Bovine diseases causing neurological signs and death in Mexican feedlots

**DOI:** 10.1007/s11250-014-0572-y

**Published:** 2014-03-27

**Authors:** Rafael Ramírez-Romero, Cecilia Ramírez-Hernández, Luis Jorge García-Márquez, Rafael Julio Macedo-Barragán, Julio Martínez-Burnes, Alfonso López-Mayagoitia

**Affiliations:** 1 Facultad de Medicina Veterinaria y Zootecnia, Campus de Ciencias Agropecuarias, Universidad Autónoma de Nuevo León, Av. Francisco Villa s/n, Ex Hacienda el Canadá, 66050 Gral. Escobedo, NL México; 2 Centro Universitario de Investigación y Desarrollo Agropecuario, Universidad de Colima, Km 40, Autopista Colima-Manzanillo, 28100 Tecomán, Col México; 3 Facultad de Medicina Veterinaria y Zootecnia, Universidad de Colima, Km 40, Autopista Colima-Manzanillo, 28100 Tecomán, Col México; 4 Facultad de Medicina Veterinaria y Zootecnia, Universidad Autónoma de Tamaulipas, 87000 Cd Victoria, Tamps México; 5 Atlantic Veterinary College, University of Prince Edward Island, 550 University Ave, Charlottetown, PE C1A 4P3 Canada

**Keywords:** Feedlot cattle, Bovine paralytic rabies, Botulism, Thrombotic meningoencephalitis, Polioencephalomalacia, Mexico

## Abstract

The number of large feedlot operations, similar to that of USA and Canada, has notably increased in Mexico in the last three decades. Clinical and laboratory diagnoses of neurological diseases in feedlot cattle are crucial in Mexico and Central America because of the high incidence of bovine paralytic rabies (BPR). Because of its zoonotic potential, BPR must be promptly diagnosed and differentiated from other bovine neurological diseases such as thrombotic meningoencephalitis (TME), polioencephalomalacia (PEM) and botulism. More recently, BPR and botulism have been diagnosed with increasing frequency in Mexican feedlots. Neither BPR nor botulism has relevant gross lesions, thus post-mortem diagnosis without laboratory support is impossible. Herein, we describe five outbreaks of neurological diseases in Mexican feedlots in which BPR, botulism and PEM were diagnosed either independently or in combination. A diagram illustrating the most conspicuous pathologic findings and ancillary laboratory test required to confirm the diagnoses of these neurological diseases in feedlot cattle is proposed.

## Introduction

**Fig. 1:**
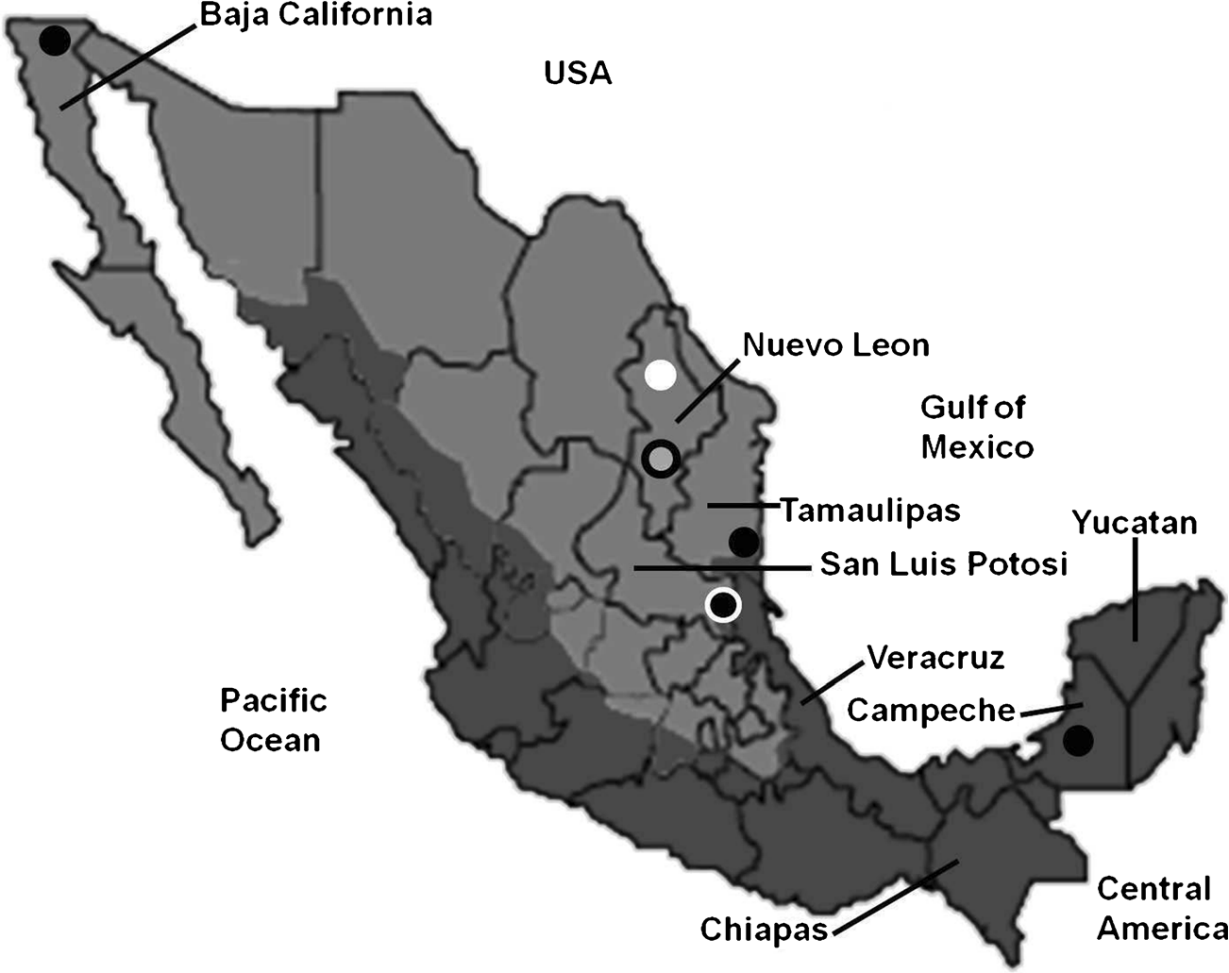
Map of Mexico. BPR control zones are depicted in *darker colour*; these zones harbour vampire bats. Those states included have been referred in the text. *Black dots* are confirmed with BPR outbreaks (Note: the outbreaks in Aldama and Mexicali are not described in text but included in map). The *white dot* corresponded to a confirmed botulism outbreak (outbreak 4, Sabinas Hidalgo, Nuevo León). The *black dot with a white outline* refers to a BPR outbreak (outbreak 2, Tamuin, San Luis Potosi) and to other different outbreak occurring in the same area in which there were both BPR and botulism (outbreak 3 Tamuin, San Luis Potosi). The *gray dot with a black outline* corresponds to the last outbreak, which is a concomitance of both PEM and BPR (outbreak 5, General Escobedo, Nuevo León). The map was modified from the original source: FAO/SAGARPA 2007

Bovine paralytic rabies (BPR) is endemic in many geographical areas of Mexico, particularly in those which are normal habitat for vampire bat (*Desmodus rotundus*). These high-risk areas include zones along the Gulf of Mexico and the Pacific Coast (Fig. 1) (FAO/SAGARPA 2007; Lee et al. 2012). Several outbreaks of vampire-transmitted rabies have been described in Aldama, Mexico (Fig. 1, Tamaulipas) (Martinez-Burnes et al. 1997; Ramírez Romero et al. 2011).

Mexico has extensive grass lands which are suitable to raise beef cattle (Huntsinger and Starrs 2006). At 2007, Mexico registered an inventory of 23 million bovines of which almost 19 million were beef cattle (Peel et al. 2011). Nonetheless, the inventory is actually declining. Over a million heads are exported yearly to the USA, mostly from the northern Mexico close to the Mexican-American border (Huntsinger and Starrs 2006). Crossbreed beef cattle with minimal zebu blood are preferred by USA importers, while zebu crosses are typically used for domestic consumption in Mexico (Peel et al. 2011). The vast majority of animals fattened in northern Mexico feedlots originate from tropical and subtropical regions of Mexico and Central America where vampire bats and BPR are endemic (Peel et al. 2011; Lee et al. 2012).

In 2011, 148 confirmed cases of animal rabies were reported in Mexico, most of them (82 %) occurred in cattle (Blanton et al. 2012). Interestingly, a cow imported from Mexico was diagnosed with rabies in Texas, USA, and the virus involved was identified as vampire bat strain probably originated from the border state of Tamaulipas, Mexico (Fig. 1, Tamaulipas) (Blanton et al. 2011). Three confirmed cases of BPR have also been reported in a feedlot in the border town of Mexicali in Baja California, Mexico, a geographical location far distant from the rabies-endemic areas (Fig. 1). Baja California is an arid territory unsuitable to be inhabited by vampire bats (Lee et al. 2012).

The risk of moving cattle from endemic to BPR-free areas has been underestimated, and it is not until recently that the importance of BPR in feedlots of northern Mexico has been documented.

## Representative outbreaks

During 2012 and 2013, several outbreaks of feedlot mortality in cattle showing neurological signs were investigated.

### First outbreak

The first outbreak occurred in Escarcega, Campeche (Fig. 1), where a mortality of 32.4/1,000 was reported. Prior to death, one of the affected animals showed progressive hindlimb ataxia, aggressive behaviour, prostration and tenesmus. Post-mortem examination revealed no significant gross findings, but the brain of this bullock subsequently tested positive for rabies by fluorescence antibody test (FAT). Another animal also tested positive for rabies by FAT but full necropsy was not done. Similar cases occurred in animals maintained in the feedlot and in animals on prairie. In this system, animals are grazing for at least 45 days (preconditioning), before being moved to the feedlot (finishing). Although this particular geographical area is endemic for BPR, rabies vaccination was not done until the BPR diagnosis had been made. In this outbreak, BPR most likely occurred locally where the animals were kept.

### Second outbreak

Another outbreak occurred in Tamuin, San Luis Potosi (Fig. 1), in a feedlot that also had extensive grassland areas for preconditioning. Detailed clinical and post-mortem studies were done in one animal, and the brain was collected and submitted to the laboratory from another one. These two animals were representative of the outbreak with a mortality of 28.6/1,000; most of the affected animals were recently introduced to grass (less than 12 days before). The majority of animals had been purchased in Chiapas (Fig. 1). Clinically, these animals developed progressive hindlimb ataxia and posterior hypoaesthesia, became prostrated, showed trismus and tenesmus and died. Both animals tested positive for rabies by FAT. Although vampire bats are present in this zone, the infection most likely occurred in southern Mexico prior the animals been moved to the feedlot. It is worthwhile noting that in these outbreaks of Tamuin and Escarcega, the presumptive diagnosis was originally thrombotic meningoencephalitis (TME), an infectious disease caused by the bacterium *Histophilis somni*. Hence, treatments and management of affected animals were carried out ignoring the possible risk of rabies. Veterinarians and farm personnel received post-exposure treatment after the rabies diagnosis was confirmed.

### Third outbreak

**Fig. 2:**
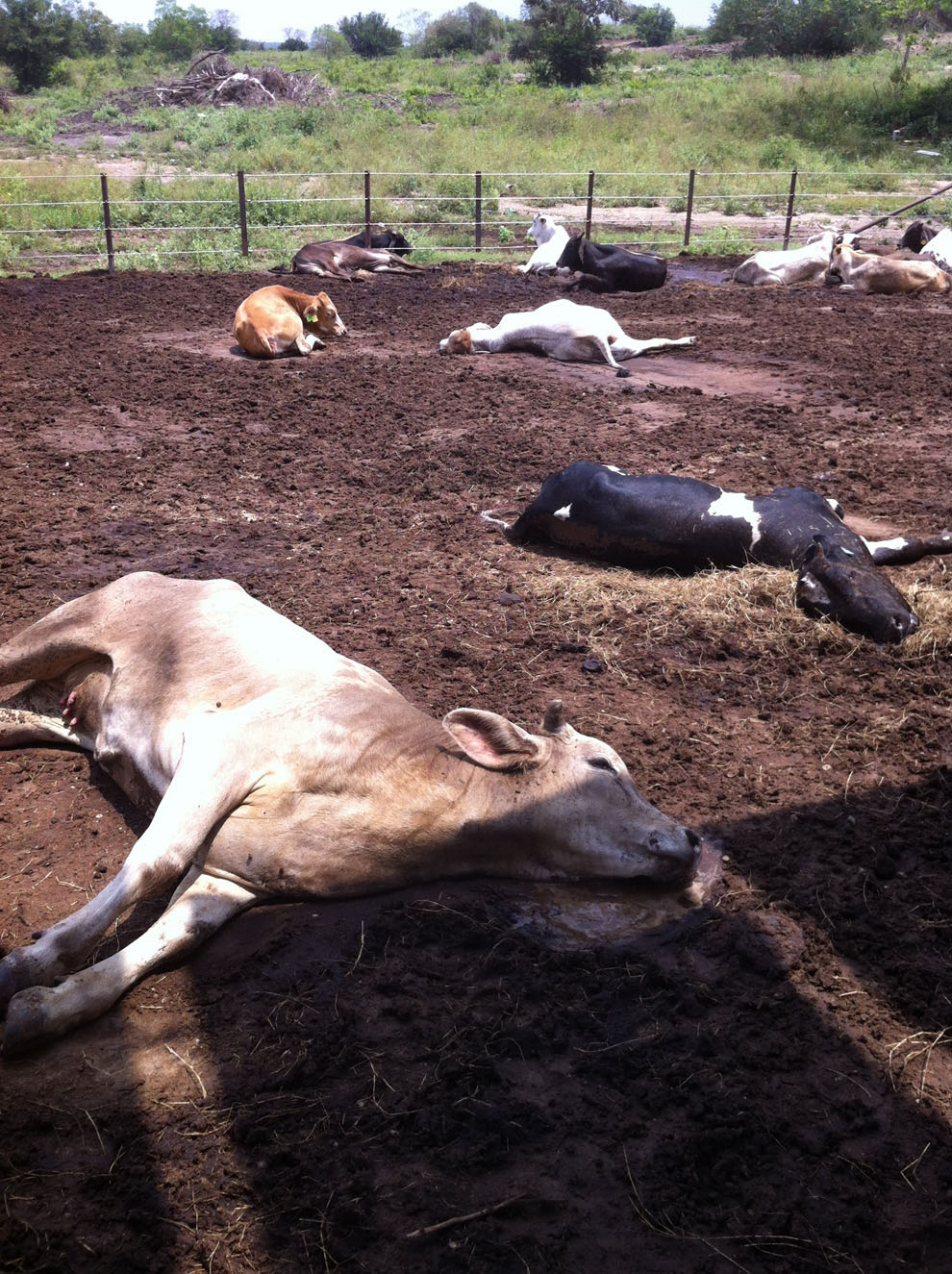
Prostrated animals from the same pen showing signs consistent with botulism. However, in this ranch, a BPR-positive case confirmed by FAT occurred previously in a grazing heifer. The zone is within the risk areas for BPR (outbreak 3, Tamuin, San Luis Potosi)

This outbreak occurred also in Tamuin, San Luis Potosi (Fig. 1), but in a different feedlot where the local veterinarian provided information regarding a previously confirmed case of BPR in a heifer that died few days after being put on a prairie. The outbreak continued, and by the time the problem was properly investigated, there were many feedlot and grazing cattle prostrated (mortality 75.2/1,000). Because of the neurological signs and previous diagnostic history in the premises, BPR was suspected in spite that it was unusual for BPR to cause recumbence in many animals at the same time (Fig. 2). Typically, cattle with BPR become ataxic and fall prostrate at different time during an outbreak, presumably due to variations in the incubation period. Most of the animals in this outbreak had no fever, continued conscious, and showed a tendency to remain in sternal recumbence with the head turned to the flank, but skin sensation remained. Necropsy was performed in two animals, and in both cases, the brain tested negative for rabies by FAT. Neither post-mortem nor histopathological examinations of the brain resulted in significant microscopic changes. Further field investigation revealed that poultry litter had been recently added to the feed. By this reason alone, botulism became the top differential diagnosis. Blood serum collected from these animals was inoculated intraperitoneally to adult mice causing the death of the rodents within 36 h. This finding was considered positive for botulism toxin, but the specific toxin type could not be identified. Additional laboratory tests confirmed that poultry litter which was added to the diet contained botulism toxin. Mortality in this feedlot stopped when the poultry litter was eliminated from diet.

### Fourth outbreak

This outbreak of feedlot mortality (62.5/1,000) appeared in Sabinas Hidalgo, Nuevo Leon (Fig. 1), where affected animals showed signs of paralysis and prostration. Sabinas Hidalgo is in an arid region unsuitable for vampire bat life, and in the state of Nuevo Leon, BPR had not yet been reported (Lee et al. 2012) (Fig. 1). The affected animals had arrived from various locations of central and south-eastern Mexico. The neurological signs were similar to those registered in the previous outbreak, but in addition, several animals lost tongue tone and showed poor tongue retraction. Post-mortem examination of three animals failed to reveal any significant gross changes. Since the FAT for rabies was negative and there were no microscopic lesions in the brain, botulism was suspected. This disease was subsequently confirmed by botulinum toxin bioassay in mice. Similar to the previous outbreak, poultry manure had been added to the diet few days before the presentation of clinical signs. The poultry litter also tested positive for botulism toxin, and mortality stopped 16 days after the poultry litter was excluded from diet.

### Fifth outbreak

The last outbreak with low mortality (18/1,000) occurred in November, 2013, in General Escobedo, Nuevo León (Fig. 1), in animals that had arrived 2 weeks earlier from the south-eastern Mexico. The clinical signs in affected animals consisted of ataxia, opisthotonus, collapse and death. The presumptive diagnosis was polioencephalomalacia (PEM), and the veterinarian in charge decided that rabies testing was unnecessary. In this feedlot, animals are received with forage during first day and are abruptly changed to a ration with 75 % processed grain and other by-products and supplement (molasses, cottonseed hulls, dried distillers’ grain, fat, and others), leaving a proportion of 25 % or less, with bad quality fibre. Formalin-fixed tissues were submitted to our diagnostic laboratory, and microscopic examination of brain revealed neuronal necrosis and severe oedema in cerebral cortex, changes that were consistent with PEM. Interestingly, one of the animals had mild non-suppurative encephalitis with gliosis and some neurons, particularly Purkinje's cells in cerebellum showed large, cytoplasmic, and eosinophilic inclusions which were considered by the pathologist pathognomonic for rabies (Negri bodies). The veterinarian and personnel handling these animals received post-exposure vaccination and became aware that testing for rabies in cattle with neurological signs should be done even in places like Nuevo Leon state, where vampire bats are not present.

## Discussion

Since most of the cattle fattened in northern Mexico originate from south-eastern regions of the country where BPR is endemic, it is likely that the sick animals that tested positive for rabies were already infected at the time of arrival. Similar scenario has been reported in Texas, USA, where a cow imported from Mexico developed rabies after arrival (Blanton et al. 2011). Therefore, BPR should always be suspected, either in feedlot or in prairie, if cattle exhibit ataxia, knuckling of the fetlocks and ascending hind leg paralysis that progress to prostration, hypoaesthesia and death. This is particularly true for animals that recently have been moved from BPR-endemic areas such as south-eastern Mexico or Central America. Likewise, BPR should be suspected in neurological cases occurring at the late phases of fattening, weather in feedlot or prairie, particularly in zones known to be the normal habitat for vampire bats. In these feedlots, animals should be vaccinated for BPR on arrival as part of the preventive and health management programme. Only because BPR has not been previously diagnosed in a bovine exploitation is not a valid reason for not suspecting or vaccinating for BPR. Outbreaks of BPR occur suddenly when otherwise healthy vampire colonies become infected and easily spread the virus (Martinez-Burnes et al. 1997; Lee et al. 2012).

Animals with BPR tend to show neurological signs and die one by one while in botulism, several animals develop clinical signs almost simultaneously. If an outbreak of neurological disease affects many animals simultaneously and poultry manure has been added to the diet, botulism should be suspected. Botulism should always be considered in cattle with neurological signs after FAT has ruled out rabies and microscopic examination of the brain had not revealed lesions. In Mexico, outbreaks of botulism typically occur when poultry litter is added to the diet. This form of botulism is much common in the Americas than that of the classic botulism seen in Africa and Australia where phosphorous-deficient cattle develop pica and eat botulism-contaminated material (Bienvenu et al. 1990; Jean et al. 1995; Fitzpatrick 2006). Bovine diets containing poultry litter have been widely used in Mexico despite of the occasional botulism outbreaks registered since the 1980s. The main reason is the low price and availability of this non-protein nitrogenous source as diet supplement for cattle and small ruminants.

TME should also be properly investigated in outbreaks of bovine neurological diseases. For instance, two fattening heifers that died after a brief presentation of neurological signs in an endemic area were tentatively diagnosed as BPR in spite the fact that the herd had been vaccinated. After FAT came back negative for rabies, TME was suspected and confirmed when microscopic examination revealed a severe suppurative meningoencephalitis with widespread vasculitis and thrombosis, the typical lesion of this disease (García-Márquez et al. 2013).

**Fig. 3:**
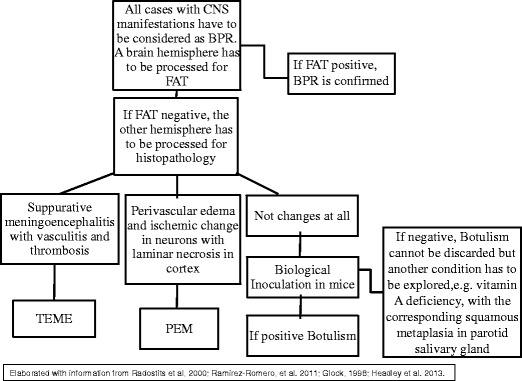
The diagram illustrates the diagnosis of BPR and ancillary laboratory tests for differential diagnosis of bovine neurological diseases

It is not possible to diagnose or differentiate clinically BPR from botulism. For instance, during an outbreak of neurological disease in Trinidad, botulism was first suspected, yet BPR was subsequently proven to be the cause (Carneiro 1954). Conversely, in an outbreak of bovine neurological disease in Turkey, rabies was initially considered when, in fact, the problem was botulism (Senturk and Cihan 2007); it should be noted that BPR and botulism can occur simultaneously in a feedlot, as in the third outbreak described here. Also, BPR may occur as co-morbidity with PEM as described in the fifth outbreak. The clinical signs for BPR, botulism, TME and PEM are summarized in Table 1. (Radostits et al. 2000; Ramírez Romero et al. 2011; Headley et al. 2013). Additionally, an algorithm for pathologic diagnosis and some complementary procedures is proposed in Fig. 3. All of these neurological diseases, including hypovitaminosis A, are common in feedlot cattle (Glock 1998; Ramírez Romero et al. 2011; Headley et al. 2013).

**Table 1:** Clinical comparison among common diseases in feedlot characterized by neurological manifestations

Clinical signs	Disease			
	Bovine paralytic rabies (BPR)	Botulism	Thrombotic meningoencephalitis (TME)	Polioencephalomalacia (PEM)
Ataxia	Yes, progressive; most obvious in hind legs	Yes, progressive; most obvious in hind legs	Yes	Yes
Amaurosis	No	No	No	Yes
Fever	No	No	Yes	No
Loss of tongue tone	No	Yes	No	No
Trismus	Yes	No	No	No
Hypoaesthesia	Yes, hind quarters	No	No	No
Bellowing	Yes	No	No	No
Opisthotonus	No	No	Yes	Yes
Tenesmus	Yes	No	No	No
Depression	No	No	Yes	Yes
Alertness	Yes	Yes	No	No
Ptosis	No	Yes	No	No
Thiamine response	No	No	No	Yes (except in sulphur-induced polio)
Antibiotic response	No	No	Yes (early treatment)	No
Gross and microscopic lesions	No gross lesion in the brain but skin lesions caused by vampire bats; microscopically, non-suppurative encephalitis with inclusion bodies	No gross or microscopic lesions	Hemorrhages and infarcts in the brain and microscopic suppurative meningoencephalitis with vascular thrombosis	Mild brain oedema, cerebellar herniation, yellow discoloration of the cerebral cortex (only in severe cases), and microscopic necrosis of cortical neurons
Hematologic abnormalities	No	No	Yes, severe neutrophilia	No
Other laboratory tests	FAT, mice inoculation test and immunohistochemistry and virus isolation	Mice inoculation, bioassay, and demonstration of botulism toxin in feed	Bacterial culture of *Histophilus somni*	Autoflorescence of gray matter under UV light

Modified from Radostits et al. 2000; Ramírez Romero et al. 2011; Glock 1998; Headley et al. 2013

**Fig. 4:**
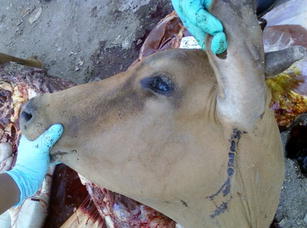
Bleeding bites inflicted by vampire bats in a confirmed case of BPR (outbreak 2, Tamuin, San Luis Potosi)

The presence of vampire wound bites in cattle with neurological signs should point out towards a possible BPR problem (Martinez-Burnes et al. 1997; Ramírez Romero et al. 2011). These wounds or scars are more commonly seen in dorsum of the ears and cervical region, among other anatomical sites (Fig. 4). In botulism, perhaps the most reliable clinical indicator is to pull and release the tongue where slow retraction suggests flaccid paralysis which is typically seen in botulism but not in BPR (Fitzpatrick 2006; Senturk and Cihan 2007).This clinical procedure proved to be useful in the diagnosis of botulism in the fourth outbreak reported here. It should be noted however that tongue retraction test is problematic to do for BPR because rabid cattle often develop trismus (“lockjaw”). Wearing gloves and taking precautions when handling animals with neurological signs are imperative since saliva is a good source of rabies virus. Contact with saliva from a rabid bovine or from tissues at a post-mortem examination of a rabid animal is an occupational risk for veterinarians, animal technicians and farmers. A fatality was reported in a veterinarian who handled a rabid ruminant in Brazil (Brito et al. 2011).

In conclusion, neurological diseases in feedlot cattle should always be properly investigated. BPR should be suspected even in northern Mexico since cattle may arrive to feedlot cattle already sick or incubating the disease. BPR should be considered in areas known to be the normal habitat for vampire bats. Preventive (pre-exposure) rabies vaccination should be mandatory for veterinarians and personnel working in Mexican feedlots. In cases where the rabies FAT results negative, other bovine neurological diseases such as PEM, TME and botulism should be investigated with the help of diagnostic laboratory. Botulism does not produce gross or microscopic lesions; thus, diagnostic is based on clinical signs, history and thoroughly investigating possible sources of botulism toxin such as poultry manure, decayed tissues or bones. Brain histopathology is a reliable diagnostic test for PEM and TME.
